# 

**Published:** 1872-09

**Authors:** 


### Books and Pamphlets Received.

---

Hysterology: A Treatise, Descriptive and Clinical, of the Diseases and Displacements of the Uterus. By Edwin N. Chapman, M. D. New York: Wm, Wood & Co., 1872. Buffalo: T. Butler & Son.

Prescription and Clinic Record. New York: Wm. Wood & Co.

Thermic Fever, or Sunstroke. By. H. C. Wood, Jr., M. D. Boylston Prize Essay. Philadelphia: J. B. Lippincott & Co., 1872. Buffalo: H. H. Otis.

The Ten Laws of Health; or, How Disease is produced and how it can be prevented. By J. R. Black, M. D. Philadelphia: J. B. Lippincott & Co. 1872. Buffalo: H. H. Otis.

The Anatomy and Development of the Rodent Ulcer. A Boylston Medical Prize Essay for 1872. By J. Collins Warren, M. D. Boston: Little, Brown & Co., 1872.

Catalogue of the Graduates and Officers of the Medical Department of the University of the City of New York. Published by the Alumni Association.

Transactions of the Medical Society of the State of Pennsylvania, at its twenty-third annual session, at Franklin, Pa., June 1872. Published by the Society.

Transactions of the Minnesota State Medical Society, held at Minneapolis, Minn., June, 1871.

Transactions of the State Medical Society of Michigan, held at Grand Rapids, June, 1872.

Transactions of the Illinois State Dental Society, held at Chicago, May, 1872.

Annual Report of the Connecticut School for Imbecils at Lakeville, Conn., 1872.

The Hallucinations of Childhood. By H. M. Knight, M. D., Lakeville, Conn. Reprinted from Proceedings of the Connecticut State Medical Society.

Report on the Museum for the Exhibition of The American Association in Philadelphia, and the Contributions from California. By Thomas M. Logan, M. D.

Medico-Legal Science. By Thad. M. Stevens, M. D., Indianapolis, Ind. Reprint from Transactions of Indiana State Medical Society.

Remarks on Leucorrhœa. By D. M. Clay, M. D., of Shreveport, La. Reprinted from American Practitioner, Aug., 1872.

The Physician and Surgeon. Vol. I. No. 1. Published monthly under the auspices of The College of Physcians and Surgeons, Baltimore.

Annual Announcements for the Session of 1872–73 of The Bellevue Hospital Medical College, New York; The Medical College of Ohio, Cincinnati, O.; St. Louis Medical College, St. Louis, Mo.; The Medical Department of The University of Pennsylvania, Philadelphia, Pa.; The Medical Department of the University of Trinity College, Toronto, Ont.; The College of Physicians and Surgeons of The Syracuse University, Syracuse, N. Y.; The Medical Department of the University of Louisiana, New Orleans, La.; The Medical Department of the University of Vermont, Burlington, Vt.; The College of Physicians and Surgeons, N. Y.; The Starling Medical College, Columbus, O.; The College of Physicians and Surgeons, Keokuk, O.; The National Medical College, Washington, D. C.; The Pennsylvania College of Dental Surgery, Philadelphia, Pa.

THE BUFFALO

**Medical and Surgical Journal**

PUBLISHED MONTHLY,

Containing Original Articles, Reports of Medical Societies and Hospitals, Editorial, Reviews, Correspondence, Army News, etc., etc.; including the usual variety of Medical Periodical Publications. Specimen copies sent on application.

Address Buffalo Medical & Surgical Journal, Buffalo, N. Y.

TERMS OF SUBSCRIPTION, - - $3.00 PER YEAR, IN ADVANCE.

